# Prevalence of suicide ideation, self-harm, and suicide among Chinese patients with schizophrenia: a systematic review and meta-analysis

**DOI:** 10.3389/fpubh.2023.1097098

**Published:** 2023-05-02

**Authors:** Yiying Liang, Manqi Wu, Yanqiu Zou, Xiaoyan Wan, Yuanyuan Liu, Xiang Liu

**Affiliations:** ^1^Department of Epidemiology and Biostatistics, West China School of Public Health and West China Fourth Hospital, Sichuan University, Chengdu, China; ^2^Department of Social Medicine and Health Management, School of Public Health, Peking University, Beijing, China; ^3^Department of Health Behavior and Social Medicine, West China School of Public Health and West China Fourth Hospital, Sichuan University, Chengdu, China

**Keywords:** schizophrenia, suicide ideation, suicide, self-harm, Chinese, meta-analysis

## Abstract

**Aims:**

Suicide ideation, self-harm, and suicide are common in patients with schizophrenia, but the reported prevalence vary largely across studies. Improved prevalence estimates and identification of moderators of the above self-directed violence are needed to enhance recognition and care, and to guide future management and research. This systematic review aims to estimate the pooled prevalence and identify moderators of suicide ideation, self-harm, and suicide among patients diagnosed with schizophrenia in China.

**Methods:**

Relevant articles published until September 23, 2021, were searched using PubMed, EBSCO, Web of Science, Embase, Science Direct, CNKI, CBM, VIP, and Wanfang databases. Eligible studies published in English or Chinese which reported the prevalence of suicide ideation, self-harm, or suicide among Chinese patients with schizophrenia were collected. All studies passed a quality evaluation. This systematic review was registered with PROSPERO (registration number CRD42020222338). PRISMA guidelines were used in extracting and reporting data. Random-effects meta-analyses were generated using the meta package in R.

**Results:**

A total of 40 studies were identified, 20 of which were evaluated as high-quality studies. Based on these studies, the prevalence of lifetime suicide ideation was 19.22% (95% *CI*: 7.57–34.50%), prevalence of suicide ideation at the time of investigation was 18.06% (95% *CI*: 6.49–33.67%), prevalence of lifetime self-harm was 15.77% (95% *CI*: 12.51–19.33%), and prevalence of suicide was 1.49% (95% *CI*: 0.00–7.95%). Multivariate meta-regression analysis revealed that age (*β* = − 0.1517, *p* = 0.0006) and dependency ratio (*β* = 0.0113, *p* < 0.0001) were associated with the lifetime prevalence of self-harm. Study assessment score (*β* = 0.2668, *p* < 0.0001) and dependency ratio (*β* = 0.0050, *p* = 0.0145) were associated with the lifetime prevalence of suicide ideation. Results of the spatial analysis showed that the prevalence of self-directed violence varied greatly across different provinces.

**Conclusion:**

This systematic review provides estimates of the prevalence of self-directed violence among Chinese patients with schizophrenia and explores its moderators and spatial patterns. Findings also have important implications for allocating prevention and intervention resources to targeted high-risk populations in high prevalence areas.

## 1. Introduction

According to WHO, approximately 800,000 people die by suicide each year (1). However, deaths from suicide represent only a small part of a larger problem because millions of people are experiencing suicidal and non-suicidal self-directed violence each year (2), which includes suicide ideation, self-harm, and suicide. Suicide ideation refers to suicidal thoughts and suicide plans (3), while self-harm refers to any type of self-injurious behavior, including suicide attempts and non-suicidal self-injury (4).

Schizophrenia is one of the top 20 causes of disability in the world (5). The relative risk of suicide is 12 times higher in patients with schizophrenia compared to patients without schizophrenia (6). It has been estimated that 10–22% of patients with schizophrenia died by suicide (7, 8), 20–40% of patients with schizophrenia make suicide attempts (9), and up to 40% of patients with first-episode psychosis experienced persistent suicide ideation (10). In addition, suicide ideation and self-harm behavior are strong risk factors for predicting subsequent deaths from suicide (11–14). It has been estimated that there are more than 10 million patients diagnosed with schizophrenia in China (15), accounting for half of this population worldwide (16, 17). Meanwhile, the suicide rate in this population is high and follows a unique pattern (8, 18), and the relative risk of suicide is 23.8(95% *CI*: 18.8–30.2) compared to the general population (8). Moreover, prevalence of suicide ideation and suicide attempts in this population are also markedly higher than those in the Chinese general population (19, 20).

Considering the severity of the problem, to understand the patterns of self-directed violence in patients with schizophrenia is of great significance for effective management and allocation of mental health resources. However, the reported prevalence varies widely, for in the original studies, the objectives, research designs, outcome measurements, target populations, and follow-up time were different. Probably due to above reasons, there are only four meta-analyses on prevalence of self-directed violence: Bai et al. (21) and Lu et al. (22) reported worldwide prevalence of suicide ideation and suicide attempts among people with schizophrenia, respectively; Fu et al. (23) reported worldwide prevalence of suicide among patients with serious mental illness. And only one meta-analysis pooled the prevalence of suicide-related behaviors in Chinese patients diagnosed with schizophrenia (20). But it only focused on suicide attempts and suicide ideation, and the experienced period of outcomes in some original studies was ambiguously defined. Meanwhile, although subgroup analyses were conducted, sources of the substantial heterogeneity among original studies were not yet clear.

To have a more comprehensive understanding of the prevalence of self-directed violence among patients diagnosed with schizophrenia in China, we conducted a systematic review study of the suicide ideation, self-harm, and suicide prevalence in this population, as well as exploring their spatial distribution. Furthermore, to achieve a more precise outcome, we defined the experienced period of self-directed violence in detail to distinguish lifetime prevalence, prevalence during hospitalization, and prevalence at the time of investigation, which also ensured the homogeneity of studies to be pooled. Finally, to explore sources of heterogeneity and possible influencing factors of each type of self-directed violence in-depth, we also added district-level indicators corresponding to the original studies in joint subgroup analysis and meta-regression. To our knowledge, this is the first systematic review focusing on the prevalence of all categories of self-directed violence among Chinese patients with schizophrenia. Conclusions of this study are expected to guide self-directed violence prevention and management strategies.

## 2. Materials and methods

This systematic review was registered with PROSPERO, identifier CRD42020222338, and was conducted according to PRISMA (24) reporting guidelines.

### 2.1. Search strategy and selection criteria

We systematically searched relevant articles on suicide ideation, self-harm and suicide among patients diagnosed with schizophrenia in China, published from inception to September 23, 2021, using both English (PubMed, EBSCO, Web of science, Embase, Science Direct) and Chinese (China National Knowledge Infrastructure, Chinese Biological Medical Literature Database, VIP Database, and Wanfang Database) databases. And we carried out a manual retrieval concurrently as supplement. The search terms used included (“suicide” or “suicidal behavior” or “suicidal plan” or “suicidal attempt” or “suicidal ideation” or “self-injury” or “self-harm” or “self-mutilation” or “self-inflicted” or “self-destruction”) and (“schizophrenia”) and (“China” or “Chinese”).

Inclusion criteria were: (1) Cross-sectional or cohort study that provided baseline data; (2) The study subjects were Chinese patients diagnosed with schizophrenia according to one or more of the following criteria: the Diagnostic and Statistical Manual of Mental Disorders (DSM), the International Statistical Classification of Diseases and Related Health Problems (ICD) or the Chinese Classification of Mental Disorders (CCMD); (3) Studies that had clear definition of suicide ideation, self-harm or suicide; (4) Prevalence was reported, or data were provided for calculation; (5) For several articles based on a same study, the one with the most comprehensive data would be included. All studies included in this study met the criteria described by the participants, interventions, comparators, outcomes, and study designs (PICOS). Participants (P): Chinese patients diagnosed with schizophrenia; Interventions (I): not applicable; Comparators (C): not applicable; Outcomes (O): reported data for suicide ideation, self-harm and suicide prevalence; and Study designs (S): all included articles were epidemiological surveys or empirical studies with prevalence data.

Exclusion criteria were: (1) Review articles or abstracts; (2) Articles with unusable data; (3) Articles that provided no definitive descriptions about surveyed regions; (4) Non-Chinese and non-English articles; (5) Articles with unclear reporting timeframe of prevalence.

### 2.2. Quality assessment and data extraction

Based on the inclusion and exclusion criteria, two investigators (Y Liang and M Wu) conducted article screening, quality assessment and data extraction independently and their disagreements were resolved by consulting a third investigator Y Liu. The methodological quality of included studies was evaluated based on the 9 criteria in The Joanna Briggs Institute Checklist for Prevalence Studies (JBI) (25): (1) sample frame; (2) recruitment procedure; (3) sample size; (4) description of participants and settings; (5) coverage of the identified sample; (6) validity of outcome measurement; (7) reliability of outcome measurement; (8) appropriateness of statistical analyses; (9) response rate. Since JBI has no cut-off criteria for classifying high-quality study, we also used another quality assessment tool (26) to ensure precision of pooled prevalence. A study was considered as being of high quality if it recruited a representative sample and if the diagnosis was validated.

For data extraction, the two investigators used a same form to extract the following information: first author, title, publication year, investigation period, survey region, diagnosis criteria, sample size, source of sample, mean age, mean illness duration, number of male/female subjects, type of events, and number of events.

In addition, considering some district-level socioeconomic indexes may play as potential moderators in meta-regression analyses, they were collected and matched for the corresponding year from China Statistical Yearbook and China Public Health Statistical Yearbook. The indexes include *per capita* GDP, dependency ratio, illiteracy ratio, unemployment ratio, the number of physicians and hospital beds per 1,000 persons. Variables (potential moderators) used in this study are listed in Supplementary Table S1.

### 2.3. Data analysis

The meta-analyses of prevalence were carried out based on the Freeman-Tukey double arcsine transformation (27) using the meta package in R 3.6.2. A pooled estimate was only calculated if there were more than 2 studies in each category of self-directed violence. Also, to ensure representativeness, pooled estimates of high-quality studies were served as the basis of our conclusions. Heterogeneity between studies was evaluated using *Q* test along with *I*^2^ statistic. *I*^2^ ≤ 50% represented relatively low heterogeneity, and meta-analysis was conducted using a fixed-effect model, while *I*^2^ > 50% represented higher heterogeneity (28), then a random effect model would be used. To further explore sources of heterogeneity, in each category with more than 4 high-quality studies, subgroup analysis and univariate meta-regression were conducted. When 2 or more moderators significantly accounted for heterogeneity, a multivariate meta-regression was conducted. The adjusted *R*^2^ statistic was utilized to represent the variance accounted for by the model (29, 30). The sensitivity analysis was carried out by excluding articles one by one. The publication bias was evaluated by funnel plot and Egger’s test in each category with more than 10 high-quality studies. All tests were two sided, and their significant level was set at 0.05.

In order to further explore the spatial distribution, pooled estimates on provincial level were calculated to produce heat maps using the ggplot2 package in R. Given China’s large regional variations in economic development and social-cultural characteristics, we stratified and categorized studies according to geographical characteristics (coastal/inland province), economic circles (inside/outside).^1^ As the time span of the included research is large, the spatial analyses were further stratified, taking the time changes in policy and economic factors into account. From 2002, China attached increased importance to mental health and the number of issued documents generally showed an increasing trend (31). “China mental health work plan (2002–2010)” issued in April 2002 is the most important one. Therefore, in the following spatiotemporal analyses, the included studies were further divided into two subgroups: whether the survey year was before 2002 (including 2002) or after 2002. Studies conducted in Hong Kong, Macau and Taiwan were excluded, where economic circles were not applicable. Subsequently, pooled estimations in different groups were compared. Since in the section of spatial analysis, we aimed to provide a more comprehensive picture of the regional, epidemiological situation of self-directed violence, datasets of heat maps, and stratification analyses were not restricted to high-quality studies.

## 3. Results

### 3.1. Identification and description of studies

**Figure 1:**
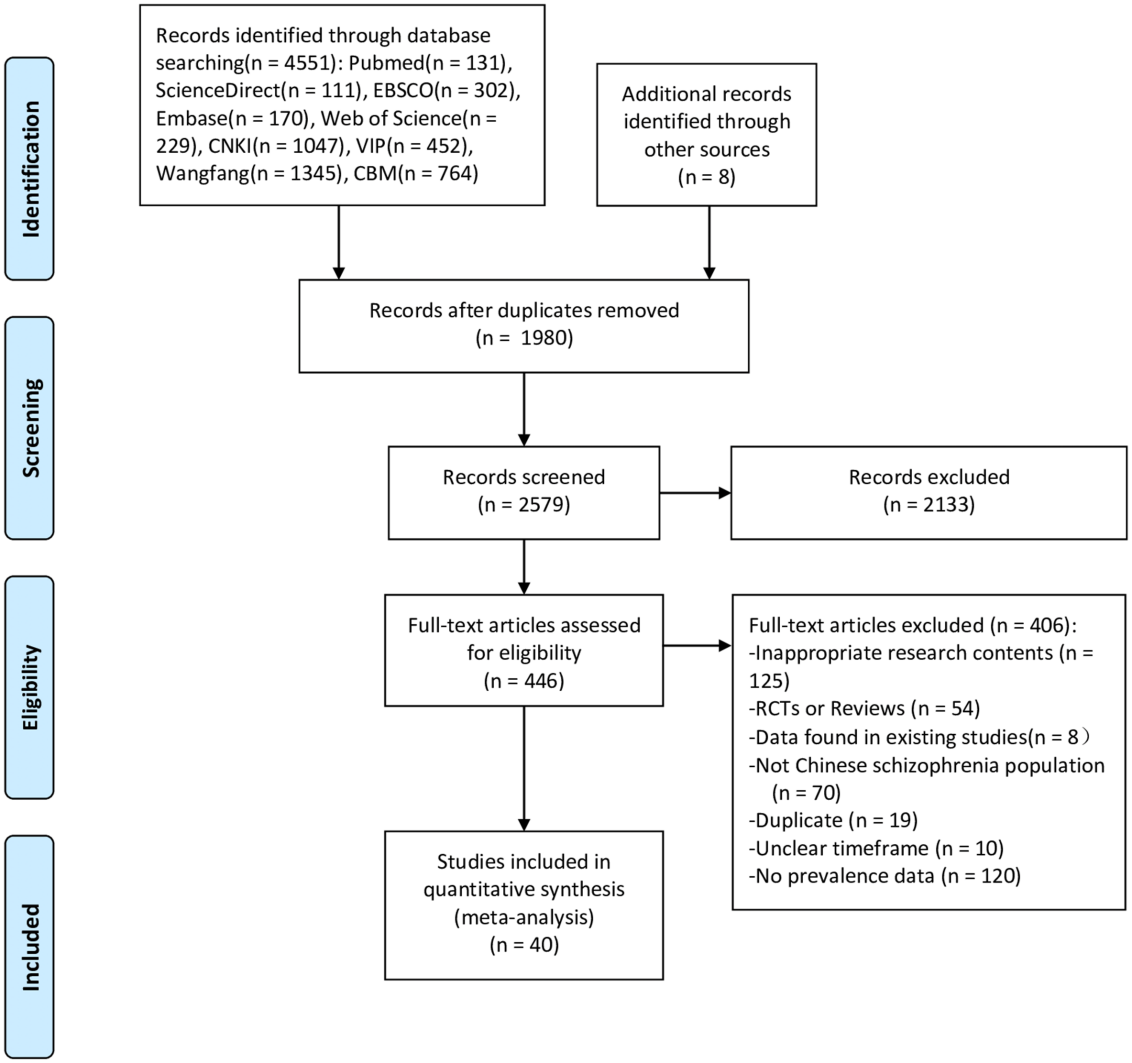
Flowchart of the selection of studies.

There were 4,559 articles identified. After reviewing titles, abstracts and full text, 40 studies (32–71) were finally selected for analyses. A PRISMA flowchart demonstrating the detailed selection process is shown in Figure 1.

In the 40 studies, 24 articles were published in Chinese and 16 were in English. A total of 140,458 patients with schizophrenia were included in these studies, whose mean age ranged from 21.9 to 65.1. There were 13 studies providing data about the prevalence of suicide ideation, ranging from 6.35 to 57.62%; 33 studies providing data about the prevalence of self-harm, ranging from 4.17 to 56.00% and 6 studies providing data about the suicide mortality, ranging from 0.18 to 4.20%. More information about included studies is shown in Table 1.

**Table 1:** Summary of the studies included in the meta-analysis.

Publication year	First author	Survey period	Study design	Province	Inside or outside the economic circles	Coastal or inland region	Source of sample	Sample size	Male/female	Mean age (years)	Mean illness duration (years)	Outcomes	Reporting timeframe of prevalence	Diagnosis Criteria of schizophrenia	Study quality
2021	Huang Y.Y et al.	2019.2–2019.9	Cross-sectional	Guangdong	inside	coastal	inpatients	426	280/146	55.34	NR	Self-harm	Lifetime	DSM-IV	6
2020	Hui L et al.	NR	Cross-sectional	Beijing	inside	inland	inpatients	731	615/116	NR	>22.75	Self-harm	lifetime	DSM-4	6
2020	Miller B.J et al.	2018.5–2018.12	Cross-sectional	Anhui	mixed	inland	inpatients	328	196/132	45.1	19.1	Self-harm, ideation	SH:lifetime SI:2 weeks	ICD-10	5
2020	Yin Y et al.	NR	Cross-sectional	Beijing	inside	inland	inpatients	159	76/83	27.1	NR	Self-harm, ideation	SH:lifetime SI:lifetime	DSM-4	6
2020	Dai Q.L et al.	2006.12–2008.12	Cross-sectional	Beijing/Hebei	inside	mixed	inpatients	908	742/166	47.8	24.3	Self-harm	lifetime	DSM-4	6
2020	Wang W.J et al.	2019.1–2019.4	Cross-sectional	Hubei/Guangdong	mixed	mixed	inpatients	627	395/232	47.72	22.0	Self-harm	lifetime	DSM-4	6
2019	Zhong Y. et al.	2018.5–2018.12	Cross-sectional	Anhui	mixed	inland	inpatients	315	190/125	45.1	19.0	Self-harm, ideation	SH:lifetime SI:lifetime, current	ICD-10	6
2019	Chang Q.S et al.	2014.1.1–2018.10.31	Cross-sectional	Guangdong	inside	coastal	inpatients	263	60/203	65.12	18.3	Self-harm, ideation	SH:lifetime SI:1 month	ICD-10	6
2019	Yue S.P et al.	NR	Cross-sectional	Sichuan	outside	inland	outpatients	358	189/169	NR	<14.93	Self-harm	Lifetime	ICD-10	5
2016	Cui Y.X	2015.1–2016.1	Cross-sectional	Shandong	outside	coastal	inpatients	100	51/49	36.1	NR	Self-harm	hospitalization	CCMD-3	2
2015	Tang X. et al.	2005.8.1–2014.12.31	Cross-sectional	Yunnan	outside	inland	outpatients	121,529	70,755/50774	NR	NR	Suicide	NA	ICD-10	5
2015	Zheng W. et al.	2013	Cross-sectional	Beijing	inside	inland	inpatients	94	48/46	37.2	10.8	Ideation	Lifetime	DSM-4	5
2014	Cui J.Y et al.	2003.1–2003.3	Cross-sectional	Beijing	inside	inland	inpatients	341	238/103	49.3	24.7	Self-harm	Lifetime	DSM-4	5
2014	Xia Y.X et al.	2010.1–2013.6	Cross-sectional	Jiangsu	inside	coastal	inpatients	48	35/13	35.12	24.8	Self-harm	Hospitalization	CCMD-3	1
2014	Qu X.L et al.	2010.8–2012.11	Cross-sectional	Xinjiang	outside	inland	inpatients	228	148/80	NR	NR	Self-harm	Hospitalization	CCMD-3	4
2013	Zhang X.Y et al.	NR	Cross-sectional	Beijing	inside	inland	inpatients	520	346/174	49.4	24.8	Self-harm	Lifetime	DSM-4	6
2013	Yan F. et al.	NR	Cross-sectional	Beijing	inside	inland	outpatients	540	267/273	42.8	17.1	Self-harm, ideation	SH:lifetime SI:current	DSM-4/ ICD-10	7
2013	Zhang J.L et al.	2011.10.01–2012.03.31	Cross-sectional	Guangdong	outside	coastal	inpatients	426	264/162	NR	NR	Self-harm	Lifetime	CCMD-3	2
2012	Kao Y.C et al.	NR	Cross-sectional	Taiwan	NA	coastal	outpatients	102	50/52	39.47	16.2	Self-harm, ideation	SH:lifetime SI:current	DSM-4	5
2012	Huang L.H	2011.01–2011.12	Cross-sectional	Jiangsu	inside	coastal	inpatients	142	142/0	29.12	NR	Self-harm	Hospitalization	CCMD-3	1
2012	Yan H.F et al.	NR	Cross-sectional	Liaoning	outside	coastal	outpatients	210	82/128	38.45	15.5	Ideation	Lifetime	DSM-4	1
2009	Lui S. Y	2002.7–2006.6	Cohort	Hong Kong	NA	coastal	outpatients	234	130/104	21.9	NR	Self-harm, suicide	Lifetime	ICD-10	4
2009	Wen L. et al.	1994.8–2006.5	Cross-sectional	Henan	outside	inland	inpatients	56	29/27	NR	NR	Self-harm	Hospitalization	CCMD-3	1
2008	Xiang Y.T et al.	2005.1–2006.6	Cross-sectional	Hong Kong	NA	coastal	outpatients	255	122/133	42.33	NR	Self-harm	lifetime	DSM-IV	8
2008	Yu J.H et al.	1998.1–2007.12	Cross-sectional	Jiangxi	outside	inland	inpatients	3,460	NR	37.2	NR	Suicide	NA	CCMD-3	2
2007	Ran M.S. et al.	1994–2004	Cohort	Sichuan	outside	inland	outpatients	500	233/267	44.7	12.5	Suicide	NA	ICD-10	5
2006	Wu B.B et al.	2003.6–2004.9	Cross-sectional	Shanghai	inside	coastal	inpatients	96	39/57	NR	NR	Self-harm, ideation	SH:lifetime SI:lifetime	CCMD-3	1
2005	Feng Y.P et al.	2003	Cross-sectional	Jiangsu	inside	coastal	inpatients	258	169/89	21.9	8.7	Ideation	Lifetime	CCMD-3	2
2004	Ran M.S. et al.	2002.5.1–2002.8.20	Cross-sectional	Sichuan	outside	inland	inpatients	145	74/71	32.2	6.6	Self-harm, ideation	SH:lifetime SI:lifetime	DSM-4	5
2004	Tang Q.F et al.	2003.12	Cross-sectional	Jiangsu	inside	coastal	inpatients	210	135/75	NR	5.7	Self-harm	Hospitalization	CCMD-3	1
2004	Qian J. et al.	2002.1–2003.3	Cross-sectional	Yunnan	outside	inland	inpatients	84	62/22	NR	NR	Self-harm	Hospitalization	CCMD-3	1
2003	Ran M.S. et al.	1994–1996	Cross-sectional	Sichuan	outside	inland	outpatients	510	239/271	NR	NR	Self-harm	Lifetime	ICD-10	5
2003	Li H.L et al.	1970.9–2002	Cross-sectional	Fujian	outside	coastal	inpatients	386	303/83	42.4	NR	Self-harm, suicide	Hospitalization	CCMD-3	2
2002	Wu D.M	2000.1–2002.1	Cross-sectional	Henan	outside	inland	inpatients	532	281/251	NR	NR	Self-harm	hospitalization	CCMD-2-R	5
2002	Zhang H.S et al.	1999.12.1–2000.2.28	Cross-sectional	Hubei	outside	inland	inpatients	177	107/70	32.36	NR	Self-harm, ideation	SH:lifetime SI:lifetime	CCMD-2-R	2
2000	Deng Z.H et al.	NR	Cross-sectional	Jiangsu	inside	coastal	inpatients	60	30/30	NR	NR	Self-harm	Lifetime	CCMD-2-R	1
2000	Hu F.S et al.	1997.8–1999.8	Cross-sectional	Shandong	outside	coastal	inpatients	73	NR	NR	NR	Ideation	Lifetime	CCMD-2-R	1
1998	Wang C.M et al.	1994.5–1997.4	Cross-sectional	Zhejiang	outside	coastal	inpatients	387	196/191	NR	NR	Self-harm	Lifetime	CCMD-2	2
1996	Liu X.W et al.	1988–1992	Cross-sectional	Heilongjiang	outside	inland	inpatients	831	NR	NR	NR	Self-harm	Hospitalization	CCMD-2	1
1996	Wang P.	1970–1987	Cross-sectional	Anhui	outside	inland	inpatients	3,605	2114/1491	NR	NR	Self-harm, ideation, suicide	SH:lifetime SI:lifetime	CCMD-2	1

NR, not reported; mixed, study conducted in mixed areas; NA, not applicable; SH, self-harm; SI, suicide ideation.

### 3.2. Meta analyses

As shown in Table 2, results of meta-analyses were stratified by study quality. Of the 40 studies included, 20 studies were classed as high quality. Based on high-quality studies, lifetime prevalence of suicide ideation was 19.22% (95%*CI*: 7.57–34.50%), prevalence of suicide ideation at the time of investigation was 18.06% (95%*CI*: 6.49–33.67%), lifetime prevalence of self-harm was 15.77% (95%*CI*: 12.51–19.33%), and suicide mortality was 1.49% (95%*CI*: 0.00–7.95%). The pooled prevalence of self-harm during hospitalization wasn’t calculated, since it was reported only in one high-quality study. In general, unrestricted pooled estimates and high-quality pooled estimates showed no significant difference. Forest plots for these outcomes are displayed in Supplementary Figures S1–S5.

**Table 2:** Pooled estimates of prevalence of suicide ideation, self-harm and suicide.

Outcomes	Period experienced	Unrestricted					High-quality studies				
		Number of datapoints in meta-analysis	Schizophrenia population sample size	Pooled prevalence (95%*CI*)	*I*^2^ (95%*CI*)	*p* value^a^	Number of datapoints in meta-analysis	Schizophrenia population sample size	Pooled prevalence (95%*CI*)	*I*^2^ (95%*CI*)	*p* value^a^
Suicide ideation^b^	Lifetime	10	5,132	26.75% (15.35–39.95)	98.2% (97.7–98.7)	<0.0001	4	713	19.22% (7.57–34.50)	95.1% (90.5–97.5)	<0.0001
	At the time of investigation	3	957	18.06% (6.49–33.67)	96.3% (92.1–98.2)	<0.0001	3	957	18.06% (6.49–33.67)	96.3% (92.1–98.2)	<0.0001
Self-harm^b^	Lifetime	23	11,513	15.40% (12.91–18.06)	92.3% (89.7–94.2)	<0.0001	16	6,528	15.77% (12.51–19.33)	92.9% (90.1–95.0)	<0.0001
	During hospitalization	10	2,617	17.27% (9.61–26.54)	96.6% (95.2–97.6)	<0.0001	1	532	10.53%	-	-
Suicide^b^	-	6	129,687	1.36% (0.57–2.45)	96.3% (94.0–97.7)	<0.0001	2	122,029	1.49% (0.00–7.95)	98.2% (95.8–99.2)	<0.0001

- Denotes no sufficient data or not available.

a Cochran’s Q test *p* value.

b Outcomes for which forest plots are available in the supplement (Supplementary Figures S1–S5).

Sensitivity analysis results did not witness significant change after excluding any study, suggesting that overall results of meta-analyses were quite stable. The funnel plot of lifetime prevalence of self-harm (Supplementary Figure S6) indicated slight asymmetry, and Egger’s test revealed a publication bias (*t* = 2.643, *p* = 0.019).

### 3.3. Sources of heterogeneity

Heterogeneity remained substantial in high-quality studies. Being limited by the number of included studies, subgroup analysis was only conducted on lifetime prevalence of self-harm and suicide ideation. Studies were classified into subgroups according to gender, source of sample, illness duration, marital status, smoking status, and survey areas. Results of subgroup analysis are shown in Supplementary Table S2. Differences between all subgroups were not significant.

Since results of subgroup analysis showed great heterogeneity among studies within each subgroup(*I*^2^>50%), we further performed meta-regression analyses, including sample size, survey year, proportion of male subjects, age, study assessment score, illness duration, and some socio-economic indexes. Univariate meta-regression results (Supplementary Table S3) revealed that illness duration (*β* = − 0.1447, *p* = 0.0052), sample size (*β* = − 0.0003, *p* = 0.0067) and age (*β* = − 0.1248, *p* = 0.0231) were negatively associated with lifetime prevalence of self-harm, while dependency ratio (*β* = 0.0074, *p* < 0.0001) and unemployment ratio (*β* = 0.0695, *p* < 0.0001) were positively associated with it. Besides, sample size (*β* = 0.0016, *p* = 0.0063) and study assessment score (*β* = 0.2822, *p* < 0.0001) were positively associated with lifetime prevalence of suicide ideation. As shown in Table 3, results of multivariate meta-regression indicated that dependency ratio (*β* = 0.0113, *p* < 0.0001) was positively associated with lifetime prevalence of self-harm, while age (*β* = − 0.1517, *p* = 0.0006) was negatively associated with it. All these moderators accounted for 89.20% of the heterogeneity. Dependency ratio (*β* = 0.0050, *p* = 0.0145) and study assessment score (*β* = 0.2668, *p* < 0.0001) were positively associated with lifetime prevalence of suicide ideation which accounted for 100% of the heterogeneity.

**Table 3:** Multivariate meta-regression models of moderators of lifetime prevalence of self-harm and suicide ideation.

Moderator Variables	Number of studies in the analysis	Coefficients(95%*CI*)	*Z* value	*p* value	$R_{adj}^{2}$
Lifetime prevalence of self-harm					
Illness duration	7	0.0941 (−0.0005 ~ 0.1887)	1.9495	0.0512	89.20%
Age		−0.1517 (−0.2380 ~ −0.0655)	−3.4477	0.0006	
Dependency ratio		0.0113 (0.0073 ~ 0.0153)	5.5874	<0.0001	
Lifetime prevalence of suicide ideation					
Study assessment score	4	0.2668 (0.1865 ~ 0.3472)	6.5068	<0.0001	100.00%
Dependency ratio		0.0050 (0.0010 ~ 0.0089)	2.4446	0.0145	

### 3.4. Spatial analyses

**Figure 2:**
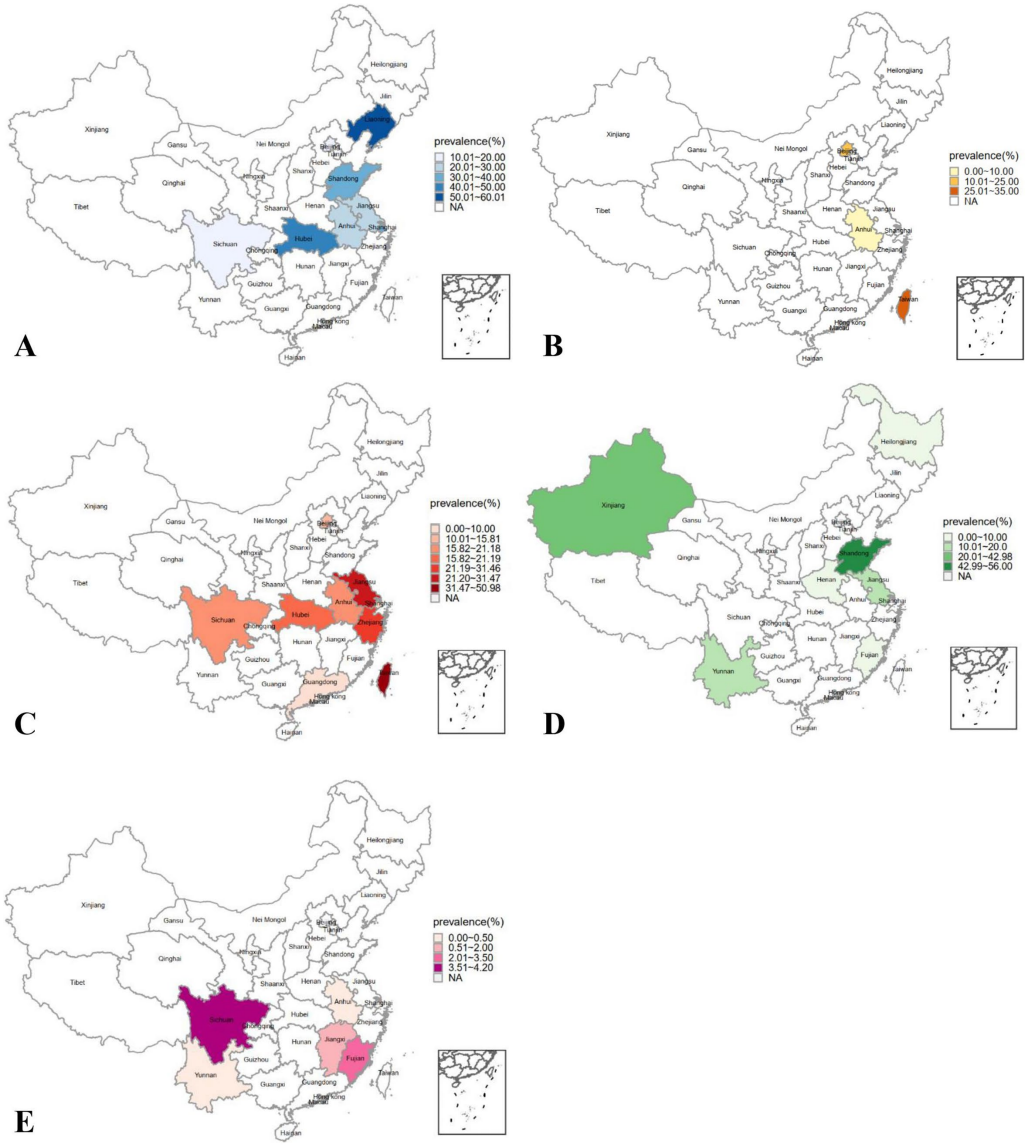
Distribution for prevalence of suicide ideation, self-harm and suicide among Chinese patients with schizophrenia. **(A)** lifetime prevalence of suicide ideation, **(B)** prevalence of suicide ideation at the time of the investigation, **(C)** lifetime prevalence of self-harm, **(D)** prevalence of self-harm during hospitalization, **(E)** suicide mortality; NA: not available.

The 40 studies included in this review were conducted in 19 provinces in China. The heat maps in Figure 2 showed that suicide ideation, self-harm, and suicide prevalence varied greatly across different regions. Except for suicide mortality, the highest prevalence of self-harm and suicide ideation occurred in coastal provinces, where some of the lowest prevalence was also identified. This suggested a large variation among coastal provinces, while results of inland provinces varied to a relatively modest extent. More details are listed in Supplementary Table S4. Subgroup analysis results showed that prevalence of suicide ideation and lifetime self-harm among patients residing within economic circles were not significantly different from patients residing outside. Similarly, differences between coastal and inland provinces were of no statistical significance (Supplementary Table S5). The spatiotemporal analysis results showed that prevalence of self-harm during hospitalization after 2002 was significantly higher than before (Supplementary Table S6). There were no significant differences in other stratified analyses of time subgroups and spatiotemporal subgroups (Supplementary Table S7).

## 4. Discussion

Being the first systematic review on the prevalence of all categories of self-directed violence among patients with schizophrenia in China, this study provides a comprehensive and detailed picture of the prevalence of suicide ideation, self-harm, and suicide prevalence in this population, and combining subgroup analysis and meta-regression to explore moderators which explain heterogeneity in depth. Findings can enhance the understanding of self-directed violence among patients diagnosed with schizophrenia and guide suicide prevention.

Suicide is a major global health issue and one of the leading causes of premature death among patients with schizophrenia (72). According to WHO, suicide mortality rate in China’s general population is 9.7/100,000 (16), which is much lower than its counterpart of 1.49% (95%*CI*: 0.00–7.95%) in this study, rendering measures for prevention essential. As for suicide mortality in patients with schizophrenia, the only previous meta-analysis review, including studies conducted in Europe, America, and Japan, demonstrates that it was 0.50% in men and 0.20% in women (73), which are also lower than our result. Possible reasons are racial differences and disparity of research time.

Historically, more attention has been paid to suicide-caused deaths instead of other self-directed violence. However, according to WHO, there are more than 20 suicide attempts for each suicide (1). Besides, self-harm behavior (74) and suicide ideation (75) are strong predictors of suicide death (2, 76, 77). Previous studies show that lifetime prevalence of suicide ideation varies largely, and a meta-analysis (19) reports that it is 3.9% (95%*CI*: 2.5–6.0%) in China’s general population. This is lower than the prevalence of 19.22% (95%*CI*: 7.57–34.50%) in our study, which reveals the severity of suicide ideation in Chinese patients with schizophrenia. In addition to general population, M.D. et al. (20) report that lifetime prevalence of suicide ideation among patients diagnosed with schizophrenia in China is 25.8% (95%*CI*: 14.7–41.1%). It is higher than our pooled result based on high-quality studies, although not significantly. However, it is similar to the unrestricted result (26.75%, 95%*CI*: 15.35–39.95%) of ours, and this is possibly due to the quality of studies included. Moreover, results of B.S. et al. (78) show that lifetime prevalence of suicide ideation is 79% in American patients diagnosed with schizophrenia, respectively, much higher than that in China. It is probably because suicide is regarded as disgraceful in Chinese culture, so respondents chose to conceal it (79). So far, few reviews have focused on suicide ideation at the time of investigation among patients diagnosed with schizophrenia, until a recent systematic review reports that it is 36.4% (95%*CI*: 30.1–43.3%) in high-income countries and 22.0% (95%*CI*: 13.3–29.0%) in low- or middle-income countries (21). Both rates are higher than the result of 18.06% (95%*CI*: 6.49–33.67%) in our study, and it may have the same reason as lifetime suicide ideation.

Although there is a wide variability in self-harm prevalence, no study has reported its pooled estimation. In this study, 17 high-quality studies provide data about self-harm, ranging from 7.45 to 50.98%, and outcomes of these studies are all suicide attempts. To date, investigations of suicide attempts have been conducted widely, and pooled lifetime prevalence of suicide attempts among patients diagnosed with schizophrenia was as high as 35.9% (95%*CI*: 29.8–42.2%) in North American population and 33.2%(95%*CI*: 27.4–37.2%) in European and Central Asian population (22). It is conceivable that lifetime prevalence of self-harm that include both suicide attempts and non-suicidal self-injury (NSSI) must be higher. Compared with these results, the lifetime prevalence of self-harm in our study [15.77% (95%*CI*: 12.51–19.33%)] is lower, also probably due to low detection rate. Besides, although NSSI has been proposed as a diagnostic entity and added in DSM5, it remains an understudied field in China that lacks notable attention (80). Therefore, to improve detection rate, more high-quality research is required.

The other focus is the high heterogeneity between studies, which may be of clinical, methodological, or statistical origin (81). In this study, design-related heterogeneity is mainly generated from the inconsistency in outcome diagnosis. One major cause of this inconsistency is the ascertainment of self-directed violence. Most studies included in this study obtained outcomes through a single question, such as: “Do you have a lifetime history of one or more suicide attempts?,” while a few studies used standardized instruments, like the Beck Suicide Ideation Scale. Both ways above guaranteed the validity of outcomes measurement because single-item measures were essentially the same as validated scales (82). However, this inconsistency in the ascertainment of self-directed violence still brought heterogeneity inevitably. The other major cause of inconsistency in outcome diagnosis is the reporting timeframe of prevalence. Miller et al. (46) and Chang et al. (32) were the only studies that utilized a 2-week and 1-month timeframe for suicide ideation, respectively. Stratification by different reporting timeframe reconciled a small degree of heterogeneity within studies, but significant heterogeneity remained.

Except for design-related heterogeneity, some moderators on individual level and district-level related to outcomes may explain heterogeneity as well. A large-scale cohort study among U.S patients with schizophrenia showed that suicide risk declined with advancing age and suggested efforts for suicide prevention focusing on young adults (83). Our study supports this idea by showing that younger patients with schizophrenia are more prone to self-harm behaviors than older patients. The other individual level moderator is illness duration, previous review showed that patients with shorter illness duration were more likely to commit self-harm than those with longer illness duration (84). This is also supported by our study, which may be due to the fact that disease is better controlled in the latter condition. As a district-level moderator, dependency ratios reveal regional, social and economic development and the degree to which local dependents are taken care of. A cross-national study confirmed that higher dependency ratios would increase psychiatric morbidity, thus leading to an increase in suicide rates (85). Our study supports this by identifying a positive association between lifetime self-harm/suicide ideation prevalence and dependency ratio. The fact that higher dependency ratios increase the burden of family caregivers may result in less care for patients diagnosed with schizophrenia. The other district-level moderator is unemployment rate. Both longitudinal study and systematic review have reported that a rise in suicide rate was linked to increasing unemployment rate in general population (86, 87), and this is also true for individuals with schizophrenia (88). Our study also confirms this relationship, suggesting that additional work stress and a sense of job insecurity among caregivers/patients may contribute to high self-harm prevalence (87, 89).

Up to now, spatial patterns of self-directed violence prevalence among Chinese patients with schizophrenia have not been reported. Studies of general population show that suicide rates vary in China by geographic location, due to regional economic and cultural differences (90, 91). Our study complements this finding by revealing that self-directed violence prevalence among patients diagnosed with schizophrenia in China varies largely across provinces. What’s more, economic disparity may contribute to the large variation, and previous studies report that patients diagnosed with schizophrenia in relatively underdeveloped economic areas have a higher risk for self-directed violence, while those in wealthier areas have a lower risk (71, 86). However, our study shows no difference in prevalence of suicide ideation and lifetime self-harm between patients residing within and outside economic circles, which may be due to the small sample size. Compared with inland provinces in China, coastal provinces have faster urbanization to drive social, economic, and occupational changes (92), which increases stress and anxiety and may lead to a higher risk of suicide ideation. This study supports this idea by presenting that lifetime prevalence of suicide ideation in coastal provinces is higher than that in inland provinces, though without statistical significance. From 2002, China attached increased importance to mental health and the number of issued documents generally showed an increasing trend, and the mortality of mental disorders in Chinese residents showed a decreasing trend (31, 93). Our study may explain its underlying mechanism by revealing that the prevalence of self-harm during hospitalization after 2002 is significantly higher than before, suggesting increased detection of self-harm during hospitalization may be effective in preventing death.

## 5. Limitations

This study has the following limitations. First, existing original studies are insufficient, thus subgroup analysis and meta-regression analysis were only performed on lifetime prevalence of self-harm and suicide ideation. Meanwhile, most of the included studies were conducted in eastern regions of China, so data from other regions were inadequate. Second, most studies did not present detailed characteristics of subjects, sources of heterogeneity could not be further analyzed. Third, in the spatial analysis, not all provinces in China were involved, and underrepresentation was caused by the single source of some provincial-level estimates. Fourth, most studies obtained outcomes retrospectively, without identifying whether it happened before or after the diagnosis of schizophrenia. Similarly, it could not be distinguished whether the self-directed violence was first-time or repeat episodes. Also, since none of the included study distinguished between suicidal self-harm or non-suicidal self-harm, data could not be split further. Finally, unpublished papers were not included in this study, so there might be some publication bias.

## 6. Conclusion

This systematic review estimates pooled prevalence and identifies moderators of suicide ideation, self-harm, and suicide among patients with schizophrenia in China. Further investigations are warranted to illuminate underlying mechanisms of self-directed violence, so as to further promote the development of mental public health in China and around the world.

## Data availability statement

The original contributions presented in the study are included in the article/Supplementary material, further inquiries can be directed to the corresponding author.

## Author contributions

YuL, MW, YiL, and XL contributed to study concept and design. YiL and YZ contributed to drafting of the manuscript. All authors contributed to the literature search, data collection, data analysis, data interpretation, and critical revision of the manuscript for important intellectual content. YiL performed statistical analyses. XL supervised the study. All authors contributed to administrative, technical, or material support. All authors contributed to the article and approved the submitted version.

## Funding

This article was supported by National Natural Science Foundation of China (grant number 81903414).

## Conflict of interest

The authors declare that the research was conducted in the absence of any commercial or financial relationships that could be construed as a potential conflict of interest.

## Publisher’s note

All claims expressed in this article are solely those of the authors and do not necessarily represent those of their affiliated organizations, or those of the publisher, the editors and the reviewers. Any product that may be evaluated in this article, or claim that may be made by its manufacturer, is not guaranteed or endorsed by the publisher.

## Footnotes

^1^The economic situation of the surveyed regions is evaluated according to whether it was included in the economic circles. As the most developed city clusters, the three economic circles are China’s core economic regions, which include the Yangtze River Delta economic circle (Shanghai and its surrounding cities), the Beijing-Tianjin-Hebei economic circle (Beijing and its surrounding cities), and the Pearl River Delta economic circle (Guangzhou and its surrounding cities).
