# Supplementary material for: Prevalence of suicide ideation, self-harm, and suicide among Chinese patients with schizophrenia: a systematic review and meta-analysis
Source: Front Public Health. 2023 May 2;11:1097098. doi: 10.3389/fpubh.2023.1097098 (PMC10186199; doi:10.3389/fpubh.2023.1097098)
Supplement: Supplementary file 1 [file Data_Sheet_1.docx]

**Supplementary Online Content**

Prevalence of suicide ideation, self-harm and suicide among Chinese patients with schizophrenia: a systematic review and meta-analysis.

Yiying Liang; Manqi Wu; Yanqiu Zou; Xiaoyan Wan, Yuanyuan Liu; Xiang Liu*

Figure S1. Forest plot of the lifetime prevalence of self-harm

Figure S2. Forest plot of the prevalence of self-harm during hospitalization

Figure S3. Forest plot of the lifetime prevalence of suicide ideation

Figure S4. Forest plot of the prevalence of suicide ideation during hospitalization

Figure S5. Forest plot of suicide mortality

Figure S6. Funnel plot of lifetime prevalence of self-harm

Table S1. Variables Assignment Table

Table S2. Subgroup analyses of the lifetime prevalence of self-harm and suicide ideation

Table S3. Univariate meta-regression of lifetime prevalence of self-harm and suicide ideation

Table S4. Provincial-level prevalence of suicide ideation, self-harm and suicide

Table S5. Spatial distribution for prevalence of suicide ideation, self-harm and suicide

Table S6. Subgroup of time for prevalence of suicide ideation, self-harm and suicide

Table S7. Subgroup of spatiotemporal characteristics for prevalence of suicide ideation, self-harm and suicide

**Figure S1. Forest plot of the lifetime prevalence of self-harm**
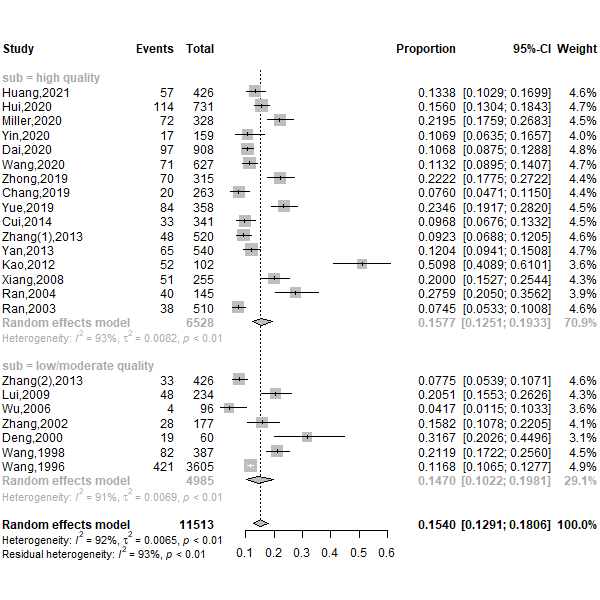


**Figure S2. Forest plot of the prevalence of self-harm during hospitalization**

**
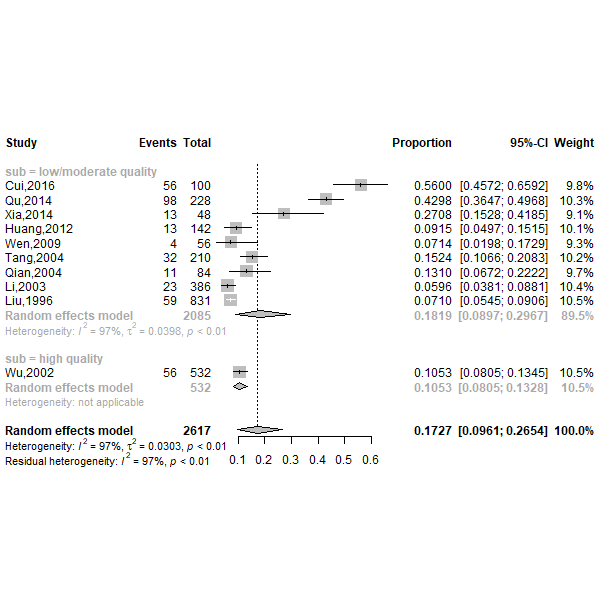
**

**Figure S3. Forest plot of the lifetime prevalence of suicide ideation**


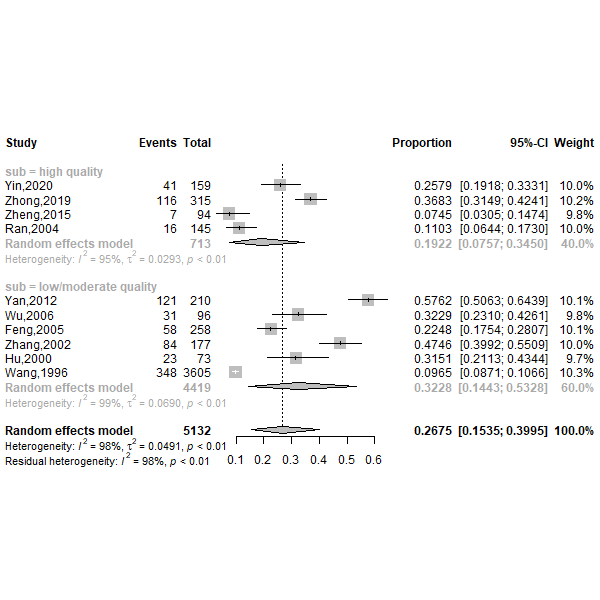


**Figure S4. Forest plot of the prevalence of suicide ideation during hospitalization**

**
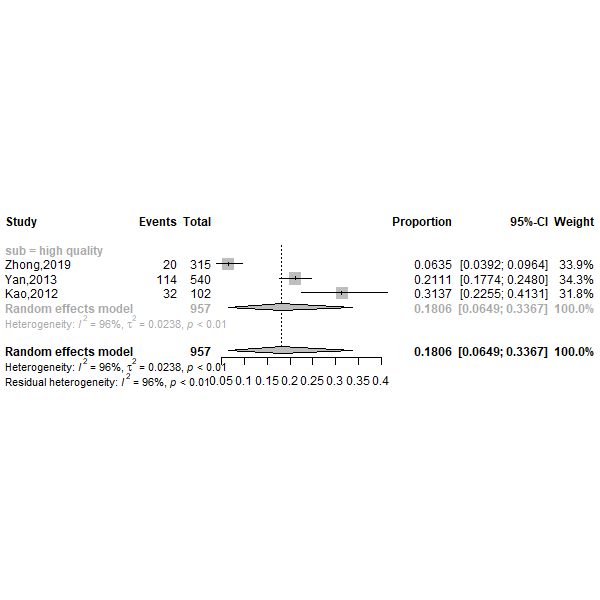
**

**Figure S5. Forest plot of suicide mortality**


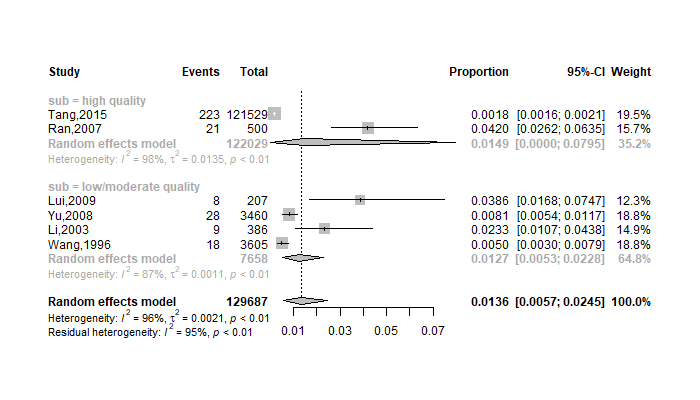


**Figure S6. Funnel plot of lifetime prevalence of self-harm**


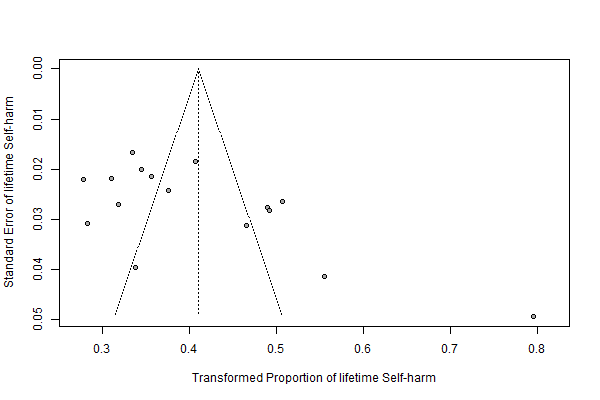


**Table S1. Variables Assignment Table**

| **Variables** | **Meaning** | **Assignment** |
| --- | --- | --- |
| Gender | Gender of subgroup population | 0: female, 1: male |
| Inside or outside the three economic circles | Whether the survey region is inside the Yangtze River Delta economic circle, the Beijing-Tianjin-Hebei economic circle, or the Pearl River Delta economic circle | 0: inside, 1: outside |
| Inland or coastal province | Whether the survey region is belonged to inland/coastal province | 0: inland, 1: coastal |
| Source of sample | The source of the sample | 0: inpatients, 1: outpatients |
| Marital status | Marital status of subgroup population | 0: married, 1: other |
| Smoking status | Smoking status of subgroup population | 0: smoker, 1: nonsmoker |
| Sample size | Sample size of the study | quantitative variable |
| Survey year | the last year of the survey period in cross-sectional study, or first year in cohort study | quantitative variable |
| Proportion of male subjects | Proportion of male patients in the study | 0:<50.0, 1:≥50.0 (%) |
| Age | Mean age of the study population | 0:<45.0，1:≥45.0 (years) |
| Study assessment score | Quality assessment score of the study | quantitative variable |
| Illness duration | mean illness duration of the study population | 0:<20.0, 1:≥20.0 (years) |
| Physicians per 1000 persons | The number of physicians per 1000 persons | quantitative variable |
| Hospital beds per 1000 persons | The number of hospital beds per 1000 persons | quantitative variable |
| Per capita GDP | Provincial Per capita gross domestic product of study regions | quantitative variable (¥100 million) |
| Dependency ratio | Total population aged ＜15 and ≥65/ total population aged 15~65 | quantitative variable (%) |
| Illiteracy ratio | The proportion of the illiterate population in the population aged 15 and above | quantitative variable (%) |
| Unemployment ratio | Unemployed population/ total population | quantitative variable (%) |

**Table S2. Subgroup analyses of the lifetime prevalence of** **self-harm and suicide ideation**

| **Classification** | **Subgroup** | **Number of studies** | **Number of cases** | **Sample size** | **Prevalence**  **(%)** | **Lower 95%*CI*** | **Upper 95%*CI*** | ***I*²**  **(%)** | ***P* value** |
| --- | --- | --- | --- | --- | --- | --- | --- | --- | --- |
| **Lifetime prevalence of self-harm** | | | | | | | | | |
| Gender | Male | 10 | 392 | 3199 | 12.98 | 9.92 | 16.38 | 85.10 | ＜0.0001 |
|  | Female | 10 | 232 | 1470 | 16.57 | 11.23 | 22.67 | 88.3 | ＜0.0001 |
| Source of sample | inpatients | 11 | 639 | 4763 | 13.87 | 11.00 | 17.01 | 88.60 | ＜0.0001 |
|  | outpatients | 5 | 259 | 1765 | 20.78 | 11.18 | 32.39 | 96.60 | ＜0.0001 |
| Mean illness duration | ≤20 years | 7 | 403 | 2051 | 19.00 | 11.68 | 27.58 | 96.00 | ＜0.0001 |
|  | ＞20 years | 5 | 363 | 3127 | 11.31 | 9.21 | 13.61 | 73.4 | 0.0046 |
| Marital status | married | 8 | 156 | 1193 | 13.05 | 8.94 | 17.76 | 79.90 | ＜0.0001 |
|  | single/divorced/deceased | 8 | 305 | 2747 | 10.97 | 9.27 | 12.78 | 49.80 | 0.0522 |
| Smoking status | smoker | 5 | 148 | 1257 | 11.40 | 9.65 | 13.27 | 0.00 | 0.5150 |
|  | nonsmoker | 5 | 154 | 1338 | 11.56 | 6.78 | 17.39 | 89.30 | ＜0.0001 |
| Inland or coastal province | inland | 10 | 637 | 4356 | 15.26 | 11.42 | 19.53 | 92.00 | ＜0.0001 |
|  | coastal | 4 | 180 | 1046 | 20.79 | 9.11 | 35.63 | 96.40 | ＜0.0001 |
| Inside or outside economic circles | inside | 7 | 403 | 3368 | 11.51 | 9.63 | 13.53 | 66.20 | 0.0070 |
|  | outside | 4 | 210 | 1533 | 15.73 | 7.69 | 25.94 | 95.80 | ＜0.0001 |
| **Lifetime prevalence of suicide ideation** | | | | | | | | | |
| Gender | Male | 3 | 39 | 314 | 12.26 | 8.77 | 16.21 | 0.00 | 0.7655 |
|  | Female | 3 | 55 | 254 | 15.28 | 1.97 | 36.89 | 93.3 | ＜0.0001 |
| Marital status | married | 2 | 36 | 130 | 23.24 | 5.83 | 47.16 | 86.2 | 0.0071 |
|  | single/divorced/deceased | 2 | 41 | 279 | 10.09 | 0.89 | 26.44 | 88.00 | 0.0040 |

**Table S3. Univariate meta-regression of lifetime prevalence of self-harm and suicide ideation**

| Moderator Variable | Number of studies in the analysis | Coefficients(95%*CI*) | *Z* value | *P* value |
| --- | --- | --- | --- | --- |
| **Lifetime prevalence of self-harm** | | | | |
| Sample size | 16 | -0.0003(-0.0005~-0.0001) | -2.7130 | 0.0067 |
| Survey year | 10 | -0.0015(-0.0081~0.0051) | -0.4500 | 0.6527 |
| Proportion of male subjects | 11 | -0.1075(-0.2145~0.0004) | -1.7444 | 0.0591 |
| Age | 13 | -0.1248(-0.2325~-0.0171) | -2.2713 | 0.0231 |
| Study assessment score | 16 | -0.0346(-0.0941~0.0248) | -1.1408 | 0.2539 |
| Illness duration | 12 | -0.1447(-0.2462~-0.0432) | -2.7943 | 0.0052 |
| Physicians per 1000 persons | 10 | -0.0108(-0.0220~0.0004) | -1.8860 | 0.0593 |
| Hospital beds per 1000 persons | 11 | -0.0087(-0.0499~0.0325) | -0.4134 | 0.6793 |
| Per capita GDP | 12 | -0.0000(-0.0000-0.0000) | -0.8813 | 0.3782 |
| Dependency ratio | 11 | 0.0074(0.0043-0.0104) | 4.7297 | <0.0001 |
| Illiteracy ratio | 12 | 0.0031(-0.0068-0.0131) | 0.6152 | 0.5384 |
| Unemployment ratio | 11 | 0.0695(0.0347-0.1043) | 3.9181 | <0.0001 |
| **Lifetime prevalence of suicide ideation** | | | | |
| Sample size | 4 | 0.0016(0.0004~0.0027) | 2.7308 | 0.0063 |
| Survey year | 3 | 0.0155(-0.0209~0.0519) | 0.8342 | 0.4042 |
| Proportion of male subjects | 3 | 1.6049(-3.0549~6.2647) | 0.6750 | 0.4996 |
| Study assessment score | 4 | 0.2822(0.1403-~0.4240) | 3.8981 | <0.0001 |
| Physicians per 1000 persons | 4 | -0.0078(-0.0463~0.0306) | -0.3988 | 0.6900 |
| Hospital beds per 1000 persons | 4 | 0.0530(-0.0604~0.1663) | 0.9160 | 0.3597 |
| Per capita GDP | 4 | 0.0000(-0.0000-0.0000) | 0.2448 | 0.8066 |
| Dependency ratio | 4 | 0.0073(-0.0109-0.0254) | 0.7870 | 0.4313 |
| Illiteracy ratio | 4 | -0.0030(-0.0443-0.0382) | -0.1437 | 0.8857 |
| Unemployment ratio | 4 | -0.0059(-0.1575-0.1457) | -0.0765 | 0.9390 |

**Table S4.** **Provincial-level prevalence of suicide ideation, self-harm and suicide.**

| **Classification** | **province** | **Lifetime prevalence of self-harm** | | **Prevalence of self-harm during hospitalization** | | **Lifetime prevalence of suicide ideation** | | **Prevalence of suicide ideation at the time of the investigation** | | **Suicide mortality** | |
| --- | --- | --- | --- | --- | --- | --- | --- | --- | --- | --- | --- |
|  |  | Prevalence (%)(95%*CI*) | datapoints | Prevalence (%)(95%*CI*) | datapoints | Prevalence (%)(95%*CI*) | datapoints | Prevalence (%)(95%*CI*) | datapoints | Prevalence (%)(95%*CI*) | datapoints |
| Coastal provinces | Taiwan | 50.98 | 1 | - | - | - | - | 31.37 | 1 | - | - |
|  | Jiangsu | 31.47 | 1 | 15.48(8.22~24.42) | 3 | 22.48 | 1 | - | - | - | - |
|  | Zhejiang | 21.19 | 1 | - | - | - | - | - | - | - | - |
|  | Guangdong | 9.50(6.10~13.53) | 3 | - | - | - | - | - | - | - | - |
|  | Shanghai | 4.17 | 1 | - | - | 32.29 | 1 | - | - | - | - |
|  | Shandong | - | - | 56.00 | 1 | 31.51 | 1 | - | - | - | - |
|  | Fujian | - | - | 5.96 | 1 | - | - | - | - | 2.33 | 1 |
|  | Liaoning | - | - | - | - | 57.62 | 1 | - | - | - | - |
|  | Hong kong | 20.24(16.79~23.94) | 2 | - | - | - | - | - | - | 3.86 | 1 |
| Inland provinces | Sichuan | 18.37(6.37~34.01) | 3 | - | - | 11.03 | 1 | - | - | 4.20 | 1 |
|  | Anhui | 18.14(10.39~27.45) | 3 | - | - | 21.54(2.26~52.75) | 2 | 6.35 | 1 | 0.50 | 1 |
|  | Hubei | 15.82 | 1 | - | - | 47.46 | 1 | - | - | - | - |
|  | Beijing | 11.51(9.07~14.20) | 5 | - | - | 15.73(2.38~37.13) | 2 | 21.11 | 1 | - | - |
|  | Xinjiang | -- | - | 42.98 | 1 | - | - | - | - | - | - |
|  | Yunnan | - | - | 13.10 | 1 | - | - | - | - | 0.18 | 1 |
|  | Henan | - | - | 9.94(7.59~12.54) | 2 | - | - | - | - | - | - |
|  | Heilongjiang | - | - | 7.10 | 1 | - | - | - | - | - | - |
|  | Jiangxi | - | - | - | - | - | - | - | - | 0.81 | 1 |

-denotes no data

**Table S5. Spatial distribution for prevalence of suicide ideation, self-harm and suicide**

| **Classification** | **Subgroup of regions** | **Lifetime prevalence of suicide ideation (95%*CI*)** | **Prevalence of suicide ideation at the time of the investigation (95%*CI*)** | **Lifetime prevalence of self-harm (95%*CI*)** | **Prevalence of self-harm during hospitalization (95%*CI*)** | **Suicide mortality (95%*CI*)** |
| --- | --- | --- | --- | --- | --- | --- |
| Economic circles | Economic circles | 21.25% (12.72-31.25) | 21.11% (17.77−24.66) | 11.65% (9.19-14.36) | 15.48% (8.22-24.42) | - |
|  | Non-economic circles | 29.47% (9.69-54.46) | - | 14.52% (10.59-18.95) | 17.76% (7.95-30.33) | 1.11% (0.41−2.10) |
| Geographical distribution | Coastal provinces | 35.66% (18.99-54.31) | - | 12.71% (7.36-19.21) | 20.16% (7.01-37.75) | 2.33% (1.03−4.11) |
|  | Inland provinces | 21.36% (9.38-36.52) | 12.89% (2.19−30.41) | 13.54% (10.80-16.53) | 14.70% (5.26-27.68) | 0.91% (0.29−1.86) |

-denotes no data

| **Subgroup of time** | **Lifetime prevalence of suicide ideation (95%CI)** | **Prevalence of suicide ideation at the time of the investigation (95%CI)** | **Lifetime prevalence of self-harm (95%CI)** | **Prevalence of self-harm during hospitalization (95%CI)** | **Suicide mortality (95%CI)** |
| --- | --- | --- | --- | --- | --- |
| Before 2002 (including 2002) | 23.02% (7.26-44.12) | - | 15.73% (10.46-21.82) | 7.79% (5.49-10.46) | 2.35% (0.47−5.49) |
| After 2002 | 23.87% (12.77-37.10) | 6.35%* | 13.09% (10.00-16.52) | 22.66% (10.60-37.53) | 0.43% (0.03−1.27) |

**Table S6. Subgroup of time for prevalence of suicide ideation, self-harm and suicide**

-denotes no data; *denotes only one available study;

| **Regions** | **Subgroup of time** | **Lifetime prevalence of suicide ideation (95%*CI*)** | **Prevalence of suicide ideation at the time of the investigation (95%*CI*)** | **Lifetime prevalence of self-harm (95%*CI*)** | **Prevalence of self-harm during hospitalization (95%*CI*)** | **Suicide mortality (95%*CI*)** |
| --- | --- | --- | --- | --- | --- | --- |
| Economic circles | Before 2002 (including 2002) | - | - | - | - | - |
|  | After 2002 | 19.71% (8.56-33.96) | - | 9.60% (7.37-12.09) | 15.48% (8.22-24.42) | - |
| Non-economic circles | Before 2002 (including 2002) | 23.02% (7.26-44.12) | - | 15.73% (10.46-21.82) | 7.99% (5.49-10.46) | 1.99% (0.19−5.41) |
|  | After 2002 | - | - | 7.75%* | 27.87% (9.52-51.14) | 0.43% (0.03−1.27) |
| Coastal provinces | Before 2002 (including 2002) | 31.51%* | - | 21.19%* | 5.96%* | 2.33%* |
|  | After 2002 | 26.58% (17.57-36.66) | - | 8.40% (5.27-12.14) | 24.99% (8.15-46.99) | - |
| Inland provinces | Before 2002 (including 2002) | 20.54% (3.99-45.25) | - | 14.35% (9.11-20.54) | 8.66% (5.59-12.31) | 1.85% (0.00−7.21) |
|  | After 2002 | 20.40% (0.91-54.64) | 6.35%* | 9.68* | 19.58% (2.63-46.13) | 0.43% (0.03−1.27) |

**Table S7. Subgroup of** **spatiotemporal characteristics for prevalence of suicide ideation, self-harm and suicide**

-denotes no data; *denotes only one available study;
